# The analysis of multilevel factors affecting adenoma detection rates for colonoscopies: a large-scale retrospective study

**DOI:** 10.1186/s12876-021-01983-3

**Published:** 2021-10-25

**Authors:** Liang Huang, Yue Hu, Shan Liu, Bo Jin, Bin Lu

**Affiliations:** 1 Department of Gastroenterology, First Affiliated Hospital of Zhejiang, Chinese Medical University, 54 Youdian Road, Hangzhou, Zhejiang China; 2 Key Laboratory of Digestive Pathophysiology of Zhejiang Province, First Affiliated Hospital of Zhejiang, Chinese Medical University, 548 Binwen Road, Hangzhou, Zhejiang China; 3 Center of Clinical Evaluation, The First Affiliated Hospital of Zhejiang Chinese Medical University, 54 Youdian Road, Hangzhou, Zhejiang China

**Keywords:** Colonoscopy, Adenoma detection rate, Quality indicator, Colorectal cancer

## Abstract

**Background:**

Adenoma detection rate (ADR) is a validated primary quality indicator for colonoscopy procedures. However, there is growing concern over the variability associated with ADR indicators. Currently, the factors that influence ADRs are not well understood.

**Aims:**

In this large-scale retrospective study, the impact of multilevel factors on the quality of ADR-based colonoscopy was assessed.

**Methods:**

A total of 10,788 patients, who underwent colonoscopies performed by 21 endoscopists between January 2019 and December 2019, were retrospectively enrolled in this study. Multilevel factors, including patient-, procedure-, and endoscopist-level characteristics were analyzed to determine their relationship with ADR.

**Results:**

The overall ADR was 20.21% and ranged from 11.4 to 32.8%. Multivariate regression analysis revealed that higher ADRs were strongly correlated with the following multilevel factors: patient age per stage (OR 1.645; 95% CI 1.577–1.717), male gender (OR 1.959; 95% CI 1.772–2.166), sedation (OR 1.402; 95% CI 1.246–1.578), single examiner colonoscopy (OR 1.330; 95% CI 1.194–1.482) and senior level endoscopists (OR 1.609; 95% CI 1.449–1.787).

**Conclusion:**

The ADR is positively correlated with senior level endoscopists and single examiner colonoscopies in patients under sedation. As such, these procedure- and endoscopist-level characteristics are important considerations to improve the colonoscopy quality.

## Introduction

Colorectal cancer (CRC) is the fourth leading cause of cancer-related mortality worldwide, accounting for approximately 694,000 deaths each year [1–3]. As such, CRC has become a major health problem worldwide. Notably, there are declining trends in the incidence and mortality rates of CRC in the United States (US) over the past several decades, which have been largely attributed to the increased use of colonoscopies for the detection and diagnosis of CRC at an early stage [1, 4]. Meanwhile, there is growing evidence that screening colonoscopies are a powerful modality for the prevention of CRC, through the detection and removal of adenomas [4]. The benefit of a screening colonoscopy is therefore highly dependent on the quality of the detection process. Thus, quality control during screening is imperative, and as such several measures have been established as indicators for screening examinations [5–7], including adenoma detection rate (ADR), cecal intubation rate (CIR), withdrawal time and bowel preparation. Of these, ADR is the most important quality indicator for colonoscopies.

Although ADR has emerged as the primary measure of mucosal inspection quality and is an independent predictor for risk of interval CRC after colonoscopy screening [8], the current guidelines raised a quality indicator of ADR of 20% or higher [5]. Hence, interventions to improve ADRs are a central focus to improve the quality of colonoscopies. However, ADRs are also known to be influenced by a number of factors, which can be classified into three categories [9]: patient-level factors (e.g. sex, body mass index (BMI), age), procedure-level factors (e.g. quality of bowel preparation, method of operation, type of equipment), and endoscopist-level factors (e.g. the level of training, experience, skill, and specialization of the colonoscopist). Although ADRs of colonoscopies can be highly variable due to these multilevel factors, it remains unclear to what extent each of these factors affect the quality of the colonoscopy. As such, empirical data to determine the effect of the combined multilevel factors on ADRs for colonoscopies is necessary.

In this study, we sought to perform a comprehensive analysis of a large-scale dataset, assessing the impact of multilevel factors on the quality of colonoscopies based on ADRs.

## Materials and methods

### Study subjects

A total of 13,495 consecutive colonoscopies performed by 21 gastroenterologists at the First Affiliated Hospital of Zhejiang Chinese Medical University between January 2019 and December 2019 were considered based on the inclusion and exclusion criteria outlined subsequently. Patients were retrospectively included in this study if they had undergone a completed colonoscopy. The patients with the following conditions were eventually excluded from this study: (1) prior colonoscopy within three years of the present study; (2) medical history of inflammatory bowel disease (IBD), CRC, or abdominal surgery; (3) fair or poor quality of bowel preparation (fair quality: some semisolid stool that could be suctioned or washed away, but > 90% of the mucosal surface visible; poor quality: semisolid stool that could not be suctioned or washed away, with < 90% of the mucosal surface visible) [10, 11]; (4) failure of cecal intubation; (5) withdrawal time under six minutes in patients without removal of polyps.

This study was conducted in accordance with the principles of the Helsinki Declaration. Due to the retrospective characteristics of the study from January 2019 and December 2019, informed consent was waived and the study was approved by the ethics committee of the First Affiliated Hospital of Zhejiang Chinese Medical University. (IRB number 2020-KL-019–01). This study has been registered at www.ClinicalTrials.gov (Trial number: NCT04397120).

### Data collection

The characteristics of all patients, physicians, and procedures were taken into account and collected. The demographic characteristics of the study patients included sex as well as age in 10-year increments (an age of 40 was set as the lower limit in the youngest group, while an age of 70 was set as the upper limit in the oldest group). Physician characteristics included sex and the level of experience of the endoscopist. For experience, two categories were defined: senior and junior endoscopist. Endoscopists were considered to be senior if they had conducted at least 3000 colonoscopies and were able to perform endoscopic submucosal dissection (ESD) independently, whereas junior endoscopists were those who had completed less than 3000 colonoscopies. Procedure characteristics included colonoscopy method (single- or dual-examiner colonoscopy [nurse-assisted]) and sedation (general anesthesia).

### Outcomes

The primary outcome was the ADR for colonoscopies. ADR was defined as the proportion of colonoscopies in which at least one adenoma was detected. All the adenoma tissues were examined histopathologically, reviewed, and confirmed by the pathologists at our center. Based on the histopathological findings, the detected adenomas were classified as either tubular, villous, tubulovillous, or serrated adenomas.

### Statistical analysis

Statistical analyses were performed under the guidance of statistician from the Clinical Evaluation and Analysis Center of the First Affiliated Hospital of Zhejiang Chinese Medical University. Continuous variables with normal distribution were expressed as the mean ± standard deviation (SD). Categorized variables were summarized as counts and proportions. Continuous variables were compared between groups using the Student’s *t*-test (normal distribution). Other categorical variables were compared between groups using the chi-squared test. A mixed-effects multivariable logistic regression model was used to determine the association between the variables of patients, endoscopists, and procedure of ADRs. All variables that were significant in the univariate analysis were included in the model. All reported *p* values were two-tailed, and a confidence interval (CI) of 95% was used throughout. A *p* value < 0.05 was considered statistically significant. All the analyses were using the statistical software SPSS version 22.0 (IBM Corporation, Somers, NY).

## Results

### Patient-level, procedure-level, and endoscopist-level characteristics

**Fig. 1:**
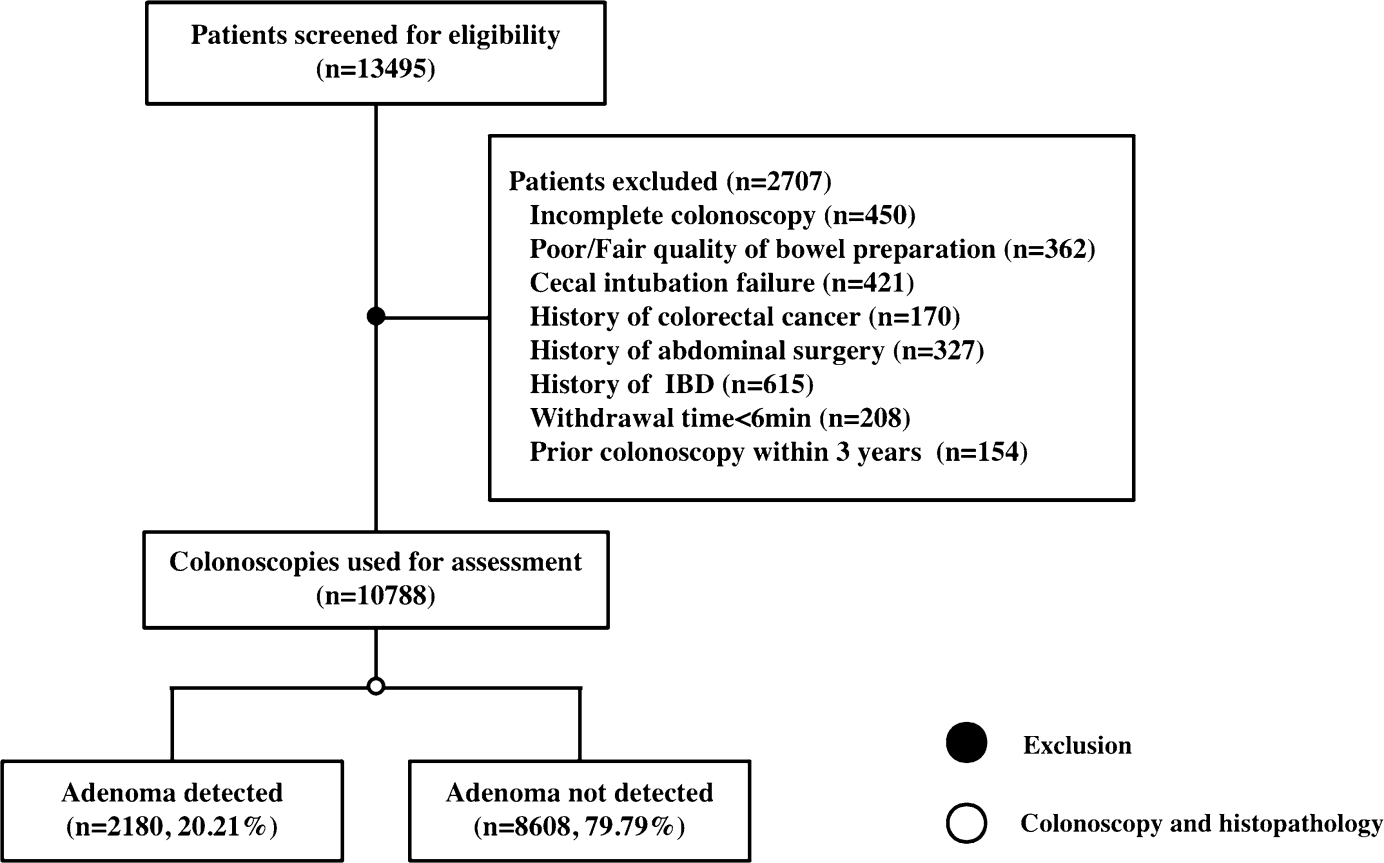
Flowchart of patient enrollment

Between January 2019 and December 2019, a total of 13,495 individuals underwent colonoscopies by 21 attending physicians or endoscopists. Among these patients, 10,788 fit the eligibility criteria and were enrolled in this retrospective analysis (Fig. 1). The patient-, procedure-, and endoscopist-level characteristics are summarized in Table 1. The mean age of patients was 52.62 ± 13.7, and the ratio of male-to-female patients was 1.04:1. During the procedure, 7581 patients (70.3%) received sedation, and single-examiner colonoscopies were performed in 7115 patients (66.0%). The 21 endoscopist s included 11 males (66.2%) and 10 females (43.8%). The endoscopists were categorized based on experience into senior level (7/21) and junior level (14/21). The overall ADR was 20.21%, and ranged from 11.4 to 32.8%. The histopathological analysis of the colorectal biopsies revealed different types of adenomas, including tubular adenomas in 1182 cases, villous adenomas in 584 cases, tubulovillous adenomas in 573 cases, and serrated adenomas in 41 cases. Notably, colorectal cancer was detected in 145 cases, accounting for 1.34% (145/10788) of the study subjects.

**Table 1:** Patient-, procedure-, and endoscopist-level characteristics

Variables	Total colonoscopy (n = 10,788) (%)	Adenoma (n = 2180) (%)	No adenoma (n = 8608) (%)	*p* value*
Sex				< 0.001
Female	5280 (48.9)	821 (15.5)	4459 (84.5)	
Male	5508 (51.1)	1359 (24.7)	4149 (75.3)	
Age, years				< 0.001
< 40	1927 (17.9)	101 (5.2)	1826 (94.8)	
40–49	2236 (20.7)	311 (13.9)	1925 (86.1)	
50–59	3100 (28.7)	702 (22.6)	2398 (77.4)	
60–69	2435 (22.6)	715 (29.4)	1720 (70.6)	
> 70	1090 (10.1)	351 (32.2)	739 (67.8)	
Mean (± SD)	52.62 ± 13.7	58.73 ± 10.91	51.08 ± 13.90	< 0.001
Sedation				< 0.001
No	3207 (29.7)	506 (15.8)	2701 (84.2)	
Yes	7581 (70.3)	1674 (22.1)	5907 (77.9)	
Colonoscopy method				< 0.001
Single-examiner	7115 (66.0)	1549 (21.8)	5566 (78.2)	
Dual-examiner	3673 (34.0)	631 (17.2)	3042 (82.8)	
Endoscopist’s experience				< 0.001
Junior	7839 (72.7)	1366 (17.4)	6473 (82.6)	
Senior	2949 (27.3)	814 (27.6)	2135 (72.4)	
Endoscopist’s sex				0.001
Female	3651 (33.8)	672 (18.4)	2979 (81.6)	
Male	7137 (66.2)	1508 (21.1)	5629 (78.9)	

*p value: Student’s *t*-test for continuous variables and χ^2^ test for categorical data

### Univariate regression analysis

**Fig. 2:**
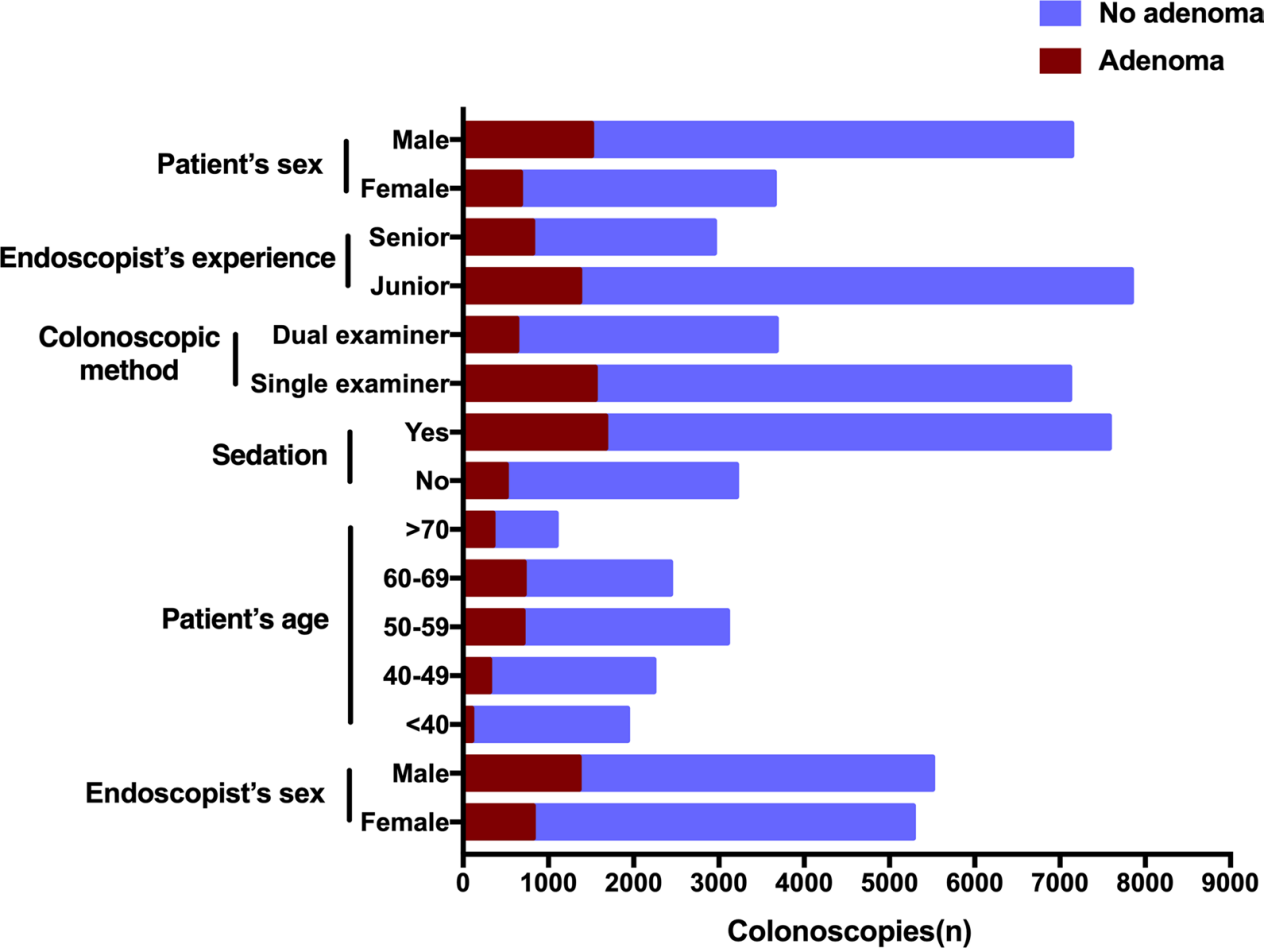
Schematic diagram of multilevel characteristics in the adenoma and non-adenoma groups

A univariate regression analysis was performed to compare patients with or without adenoma detection during the colonoscopies. As shown in Table 1, adenomas were found in 2180 patients that were assigned to the adenoma group, while the remaining 8608 patients had no adenomas and were therefore assigned to the non-adenoma group. Comparisons between the two groups were made and the results are listed in Tables 1, 2 and Fig. 2. For patient-level characteristics, we found that the proportion of male patients was significantly higher than female patients in the adenoma group (24.7% vs. 15.5%, *p* < 0.001), and that the patients in the adenoma group were significantly older than those in the non-adenoma group (58.73 ± 10.91 years vs. 51.08 ± 13.90 years, *p* < 0.001). Analysis of procedure-level factors revealed that the percentages of patients who received sedation and single-examiner colonoscopies were 22.1% and 21.8%, respectively. These values were significantly greater than the patients in the adenoma group who were not given sedation (15.8%; *p* < 0.001) and had dual-examiner colonoscopies (17.2%; *p* < 0.001). In addition, the analysis of endoscopist-level characteristics showed that a significantly greater number of colonoscopies were conducted by senior and male endoscopists in the adenoma group (senior versus junior: 27.6% vs. 17.4%, *p* = 0.000; male versus female: 21.1% vs. 18.4%, *p* < 0.001).

**Table 2:** Indicators of adenoma in the logistic regression model of colonoscopies

Variables	Regression coefficient	Crude OR (95% CI)	Adjusted OR (95% CI)	*p* value*
Sex				
Female		Reference	Reference	
Male	0.672	1.78 (1.62–1.96)	1.96 (1.77–2.17)	< 0.001
Age				
< 40		Reference	Reference	
40–49	1.020	2.92 (2.31–3.69)	2.77 (2.19–3.51)	< 0.001
50–59	1.658	5.30 (4.26–6.58)	5.25 (4.21–6.54)	< 0.001
60–69	2.027	7.51 (6.04–9.35)	7.59 (6.09–9.47)	< 0.001
> 70	2.197	8.59 (6.77–10.89)	9.00 (7.07–11.44)	< 0.001
Sedation				
No		Reference	Reference	
Yes	0.338	1.51 (1.36–1.69)	1.40 (1.25–1.58)	< 0.001
Colonoscopy method				
Single-examiner		Reference	Reference	
Dual-examiner	0.285	1.34 (1.21–1.49)	1.33 (1.19–1.48)	< 0.001
Endoscopist’s experience				
Junior		Reference	Reference	
Senior	0.475	1.81 (1.64–2.00)	1.61 (1.45–1.79)	< 0.001
Endoscopist’s sex				
Female		Reference	Reference	
Male	–	1.19 (1.07–1.31)	–	0.096

*p values refer to comparison between adenoma and nonsadenoma groups in the logistic regression analysis

### Multivariable logistic regression analysis

Multivariable logistic regression analysis was performed to determine the relationship between the multi-level factors and ADRs. As shown in Table 2, the odds ratio (OR) of adenoma detection was corrected for age, sex, sedation, colonoscopy method, and endoscopist experience level, which were all significantly related to ADR (all *p* < 0.001). Patient-level factors significantly associated with higher ADR included: age per stage (OR 1.645, 95% confidence interval (CI) 1.577–1.717; 40–49 years: OR 2.772, 95% CI 2.190–3.509; 50–59 years: OR 5.250, 95% CI 4.214–6.539; 60–69 years: OR 7.591, 95% CI 6.087–9.467; above 70 years: OR 8.995, 95% CI 7.074–11.437), and male gender (OR 1.959; 95% CI 1.772–2.166). Procedure-level factors that significantly correlated with higher ADR were as follows: sedation [OR 1.402; 95% CI 1.246–1.578], and single-examiner colonoscopy [OR 1.330; 95% CI 1.194–1.482]. The senior experience level among endoscopists was identified as a significant endoscopist-level factor with regards to higher ADRs [OR 1.609; 95% CI 1.449–1.787].

## Discussion

This comprehensive analysis of a large-scale dataset of colonoscopies has revealed the following major novel findings: (1) the overall ADR of the colonoscopies was 20.21%; (2) multilevel factors, including patient-, procedure-, endoscopist-level characteristics, had a strong impact on the ADR of colonoscopies; (3) higher ADRs were strongly correlated with older age brackets (> 40 yrs), male patients, sedation, single-examiner colonoscopies, and an attending endoscopist with a senior level of experience.

Colonoscopies have proven to be one of the best preventive measures for CRC, mainly because the procedure can be used to detect and remove adenomatous polyps that are considered precursor lesions for most CRCs. Accordingly, the ADR is highly recommended in most guidelines worldwide as a primary quality indicator for colonoscopy examinations [12]. It has been noted that colonoscopies are complicated procedures that can be influenced by a variety of factors, categorized into patient-, procedure-, and endoscopist-level. In the present study, the large-scale data were obtained from 21 attending gastroenterologists who performed 10,788 colonoscopies and detected colonic adenomas in 2180 patients. The analysis of these data revealed a strong association between the ADR and the multilevel factors.

In this study, patient-level factors potentially affecting adenoma detection by colonoscopy included patient age and sex. Our results showed a significantly higher ADR in males and elderly patients, which is consistent with the findings of previous studies [13–16]. In our cohort, the risk of colorectal adenoma detection in male patients was 1.96 times higher than in females, which may be attributed to certain unhealthy lifestyle choices in men, such as cigarette smoking [17] and alcohol consumption [18]. Meanwhile, our findings also confirmed that age remained an independent risk factor for the development of an adenoma and its detection. Our findings, along with recently published guidelines [19, 20], suggest that individuals with a family history of CRC should have their first screening colonoscopy at age 45, and individuals aged 50 years and older should undergo regular colonoscopy screenings.

For procedure-level characteristics, sedation and the colonoscopy method used during the examination were considered in our study. Previous studies have reported mixed results on adenoma detection when comparing single-examiner to dual-examiner colonoscopies [21–23]. In a recent meta-analysis [24], nurse assistance during a colonoscopy has been shown to improve the ADR, whereas a high risk of bias in randomized control trials (RCTs) has also been noted as a major limitation. In agreement with Hoff et al. [23], our study reported that single-examiner colonoscopies were associated with higher ADRs. The consistency in ratings provided by examiners can affect the reliability of the ADR. The use of a single examiner may improve the consistency in observations provided by endoscopists during the examination and enhance the patient’s comfort and compliance. Meanwhile, the degree of cooperation between two operators during dual-examiner colonoscopies may affect the detection of lesions. Furthermore, in contrast to two previous studies conducted by Bannert et al. [25] and Nakshabendi et al. [26], which showed that sedation may not increase the detection of adenomas, we found that general anesthesia during the procedure was a favorable factor to improve ADRs. Our result was not congruent with the two aforementioned studies. Considering that the cohort of patients in the present study was larger with regards to sedation, the results remain significant. We proposed that sedation-associated improvement of ADRs may be attributed to increase in patient comfort and reduction in pain levels.

In terms of endoscopist-level factors, the sex of attending endoscopists and their corresponding level of experience were evaluated in the present study. Senior endoscopists were associated with higher ADRs when compared with junior endoscopists, whereas ADRs in male and female endoscopists did not yield significant differences. It has been observed in previous studies [27, 28] that endoscopists with greater seniority and experience are able to perform higher quality colonoscopies, which includes a higher detection rate of smaller polyps and advanced histology adenomas. It may merit attention that our results show that the ADR of senior endoscopists is 10.2% greater than that of junior endoscopists. After adjusting for relevant factors in the logistic regression analysis, the differences in ADR based on the level of experience of the endoscopists remains significant. With regards to the sex of the endoscopist, a greater ADR among female endoscopists has been reported in some studies [29, 30]. On the contrary, others studies showed that female endoscopists have lower ADRs [31, 32]. In this study, although unadjusted ADRs were lower in female endoscopists, after adjusting for multiple characteristics, this difference was not statistically significant. Additionally, we noted that a large proportion of the senior endoscopists were males, which may have influenced the results in the univariate analysis.

Aside from ADR, there are other indicators for quality of colonoscopy including cecal intubation rate, quality of bowel preparation, and withdrawal time [33, 34], and these indicators can contribute to achieving higher ADRs. However, prior to conducting the comprehensive analysis in our study, we excluded colonoscopies with incomplete cecal intubation, poor bowel preparation, and short withdrawal time to avoid the potential interference from these factors.

Despite the noteworthy findings of the current work outlined above, our study may be limited in several ways. Firstly, this study was performed at a single center. Secondly, as a retrospective study, all data were collected based on information in the electronic records. As such, some patients' characteristics, such as BMI, family history, and smoking status were unavailable. Finally, no repeated colonoscopies were performed on any of the patients, which could have resulted in some adenomas being unidentified. Future prospective, multicenter studies are needed to validate the findings of the current work.

## Conclusions

This study has shown that patient-, procedure-, and endoscopist-level factors have a significant impact on the quality of colonoscopies, as reflected by ADRs. It has also been shown that the ADR is positively related to senior level endoscopists, single-examiner procedures, and sedation during the procedure. Therefore, our study has important clinical implications, and it may be beneficial to use these favorable procedure- and endoscopist-level factors to improve the quality of colonoscopies and patient care.

## Data Availability

Datasets used and analyzed during the study are available upon reasonable request from the corresponding author.

## Abbreviations

ADR: Adenoma detection rate
CRC: Colorectal cancer
CIR: Cecal intubation rate
BMI: Body mass index
IBD: Inflammatory bowel disease
ESD: Endoscopic submucosal dissection
RCTs: Randomized control trials
